# E-cigarette use and health information needs among a university student population in Melbourne, Australia

**DOI:** 10.3389/fpubh.2025.1563117

**Published:** 2025-04-09

**Authors:** Tasneem Kamoni, Melis Selamoglu, Christian Osadnik, Sanduni Madawala, Susan Kotwas, Kim Turudia, Chris Barton

**Affiliations:** ^1^Department of General Practice, School of Public Health and Preventive Medicine, Monash University, Melbourne, VIC, Australia; ^2^Department of Physiotherapy, School of Primary and Allied Health Care, Monash University, Melbourne, VIC, Australia; ^3^Respiratory Research at the Alfred Hospital, Melbourne, VIC, Australia; ^4^University Health Services, Monash University, Melbourne, VIC, Australia

**Keywords:** e-cigarette, university student, health beliefs and attitudes, health information sources, knowledge, intentions

## Abstract

**Objective:**

We explored e-cigarette use, e-cigarette knowledge, attitudes, intentions to use and access to e-cigarette health information among young adults enrolled at an Australian university.

**Methods:**

Respondents completed a survey about e-cigarette use and health resources about vaping. Data were analyzed using SPSS Version 28.0.

**Results:**

Responses were received from *n* = 1,094 students aged 18–25 years. Current e-cigarette use was reported by 13.1% of respondents, daily use 7.6% and ever use 26.8%. Prevalence was greater among men, those reporting more psychological distress, alcohol use and worse academic performance. More than half (51.2%) perceived e-cigarette use as common among their peers and one-third were curious to try an e-cigarette in the future. Domestic and international student e-cigarette use was similar, however, international students tended to access less reputable sources for health information about vaping.

**Conclusion:**

Tailored strategies for domestic and international student groups are needed to address e-cigarette use among university cohorts. Universities provide a setting in which health information and cessation support can be provided to a well-defined group, by dedicated and well-resourced health and wellbeing teams. These results provide a rich resource to guide health promotion, prevention and cessation activities on campus.

## Introduction

E-cigarette use has grown rapidly in Australia in the past 5 years. An estimated 1.5 million Australian’s reported current e-cigarette use in 2022–2023 (1), most of whom were young people aged 18–24. Young adults in Australia tend to use e-cigarettes they know contain nicotine (72%), buy them from retail stores (80%), and vape when feeling stressed or anxious (29%) (2). These trends in Australia align with global patterns (3), which indicate that younger adults have the highest likelihood of trying e-cigarettes (4).

A growing body of literature outlines health harms associated with e-cigarette use (5). Non-smokers and young people are most vulnerable to e-cigarette events and are disproportionately affected by risks such as addiction, poisoning, toxicity from inhalation, and increased smoking uptake (5). A key known harm for young people is addiction to nicotine. The effects of nicotine on the developing brain are well established (6) and there is likely a bi-directional relationship between psychological distress and nicotine use (7). Nicotine exposure during periods of active brain development has been linked to long-term cognitive and behavioral deficiencies (6). Students experiencing psychological distress may use e-cigarettes as a coping mechanism, strengthening addiction, which impacts concentration and academic performance, creating further stress. Preventing young people from using e-cigarettes to avoid developing nicotine dependence is important, as is supporting them to quit and mitigate the risk of potential long-term negative health impacts (8, 9).

Colleges and Universities are one setting where there is a large concentration of young people. There is a long standing practice of health promotion on university campuses and they are seen as important settings for health promotion and public health (10). The prevalence of ever vaping among college and university students across the US, Europe, Asia and NZ ranges from 21.2–50% (11–19). Current smoking, alcohol use, white race and gender have been identified as predictors of e-cigarette use from US samples. Studies from campuses in Europe and Asia further identify binge drinking and cigarette smoking, perceived social norms, and curiosity as potential predictors of e-cigarette use among university students.

Australian data on e-cigarette use among university students is limited. Data from one study of almost 5,000 students at the University of Queensland (UQ) reported a prevalence of ever, current and daily vaping of 20.9, 1.8% and 0.7% (20) which is well below more recent estimates of prevalence among young people (1). In the UQ study, people who used e-cigarette or tobacco cigarette were more likely to believe that e-cigarettes were less harmful, and there were important differences between domestic and international students in prevalence (higher among domestic students) and perceptions of e-cigarettes as less harmful, which has important implications for health promotion and cessation services on campus.

University campuses provide unique opportunity for health promotion and prevention activities targeting young adults through health and well-being programs. They provide students accessible youth-oriented health services many of which are free of charge. This is particularly important for international student cohorts, who are navigating an unfamiliar health system and may not have the same information and supports available to them while studying abroad. University health services need to be properly equipped to provide information on vaping and are well positioned to provide health promotion, prevention, and cessation services to students.

Considering the rapid changes in the use of e-cigarettes that have occurred in the past 5 years, the aims of this study were to (i) provide an updated estimate of the prevalence of e-cigarette use among domestic and international university students at a major Australian university; (ii) identify intentions of students to use e-cigarettes in the future related to their knowledge, attitudes and perceptions of e-cigarettes and (iii) identify preferences for accessing health information about e-cigarettes to inform future health interventions in these groups.

## Methods

### Design and setting

A cross-sectional survey was completed by young adults aged between 18 and 25 years, from Monash University in Melbourne, Australia. Monash is Australia’s largest public university by student population and approximately one in three students are enrolled as international students (21). Recruitment was primarily undertaken in person on university campuses by student peers in public spaces such as university greens, libraries and cafeteria common areas, as well as via closed university student groups and noticeboards, and at University Health Service clinics.

Participation was voluntary, not tied to any course credits or requirements, and responses were anonymous. Participants were offered the chance to enter a prize draw to win 1 of 10 gift card prizes upon completion of the survey. The response rate could not be estimated as this was a convenience sample.

### Data collection

The survey was developed using Qualtrics™ (see Supplementary material) and accessed by scanning a QR code on their smartphone, or, via links in digital advertisements. Data collection occurred between September and November 2023. We checked Internet Protocol addresses to identify and remove duplicate entries (*n* = 11) to minimize the risk of multiple entries from a single respondent.

The survey was designed specifically to appeal to young adults through the flow and design of the survey, brevity, and the use of popular culture memes and references that encouraged completion. The survey was pilot tested prior to distributing the survey with students within the Department of General Practice who matched the inclusion criteria for the study. They were asked to provide feedback on their experience including identifying any grammatical or typographical errors, flow or skip errors, and ensuring response options were appropriate for this population. Pilot testing suggested the survey could be completed in less than 5 min which was important to increase engagement and completion of the survey in this context.

The selection of items for the survey was informed by the needs of the university health services and guided by the Theory of Planned Behavior and the Health Belief Model (22–25). The TPB compromises three domains: attitudes, subjective norms and the influence of social pressure and, perceived behavioral control (26). The Health Belief Model comprises four concepts: perceived severity, perceived susceptibility, perceived benefits, and perceived barriers toward e-cigarettes (22).

#### Assessment of e-cigarette use and smoking status

Frequency of e-cigarette and smoking were classified based on the Population Assessment of Tobacco and Health study definitions (27, 28). We asked “How often do you currently vape or use e-cigarettes?” Response options included daily, at least once a week, less than weekly, not at all now but has been a regular e-cigarette user in the past, not at all now but has been an infrequent e-cigarette user in the past, or not at all and I have never been a regular e-cigarette user. We classified “Current use” as people who reported using e-cigarettes either daily, at least once a week, or less than weekly. “Past use” was classified as not using e-cigarettes at all now but regular e-cigarette use in the past or; not at all now but infrequent e-cigarette use in the past. “Never used” were respondents who had never used e-cigarettes.

We asked respondents to indicate situations they were likely to vape/use e-cigarettes with five different situations they could select, or they could select “other times” (see Supplementary material for full list).

For traditional cigarettes, we asked “How often do you now smoke cigarettes, pipes or other tobacco products (do not include e-cigarettes or vapes)?” Response options and categorization of use was the same as those for e-cigarettes. We used this information to identify dual use.

#### Assessment of e-cigarette knowledge, attitudes and beliefs, and perceived social norms

E-cigarette knowledge was assessed using five items drawn from existing e-cigarette knowledge scales (29, 30). Responses options included yes/no/unsure. These items asked about different aspects of e-cigarettes, including the content of e-cigarettes (3 items), mechanism of action of e-cigarettes (1 item), and health risks of e-cigarette use (1 item). Attitudes and beliefs (8 items) were assessed using questions from previously published scales (29, 31, 32) and were answered on a five-point Likert-scale (strongly disagree to strongly agree). We asked if participants felt vaping is common among their peer group and their concern about the use of e-cigarettes “by others in the community,” “by people they are close to,” and “own use of e-cigarettes or vaping.”

#### Intention to use e-cigarettes in the future

Susceptibility to e-cigarette initiation was assessed in people who had not used e-cigarettes. Three items, adapted for use with e-cigarette initiation as described previously (33–35) were used—“Have you ever been curious about using e-cigarettes,” “Do you think you will try an e-cigarette soon?” and “If one of your best friends were to offer you an e-cigarette, would you use it?.” Participants responded on a four-point Likert-scale ranging from “definitely not” to “definitely yes.” Respondents who answered “not at all curious” to question (i) and “definitely not” to questions (ii) and (iii) for each tobacco product were considered non-susceptible, and any other combination of responses were considered susceptible.

#### Sources of e-cigarette health information

We asked respondents to nominate whether they would access information about the health effects of e-cigarettes from nine different sources (a GP, a pharmacist, university health service, government reports/websites, websites from non-government health organizations, social media, friends or family, e-cigarette retailers, and e-cigarette manufacturers). Respondents indicated yes, no, or maybe for each source.

Finally, we asked respondents to indicate where they would advise their friend or family member to seek help if they asked for help to quit vaping (see Supplementary material for full list of response options).

#### Sociodemographic characteristics, wellbeing, and academic performance

Participants demographic characteristics, including age, gender, cultural and ethnic identification and enrolment status (domestic or international) were collected together with questions to assess psychological distress (K6) (36), alcohol use (AUDIT-C) (37) and self-reported academic performance. The six items to assess psychological distress were summed to produce a total score with a possible range of 6–30. Serious psychological distress (SPD) was defined as a score of 19 or more and has been associated with the occurrence of probable serious mental illness (36). Alcohol use frequency was categorized as less than weekly and weekly or more. Academic performance was categorized as high (self-reported weighted average mark (WAM) 70 or greater) or low (less than 70).

### Data analysis

Survey responses were downloaded to SPSS (Version 28.0) [IBM Corp. (2020) for analysis]. Frequencies were used to determine proportions of respondents using traditional tobacco products including cigarettes, pipes or other tobacco products; e-cigarette use was categorized as daily, current (defined as daily, weekly, or less than weekly), past, and never use. Flavors and type of pods used, whether students believed they contained nicotine, and the situations they were most likely to use e-cigarettes are summarized.

Chi-squared tests and Analysis of Variance (ANOVA) were used to explore differences in sociodemographic characteristics, SPD, alcohol use frequency, and WAM, between student’s e-cigarette use daily or current, and past or never use. Differences in the settings that domestic and international students used e-cigarettes were compared using chi-squared tests. Independent samples *t*-tests were used to test differences in knowledge scores.

We compared attitudes and beliefs, perceived behavioral control and perceived norms for accessing e-cigarette health information between current use and never used with logistic regression, controlling for socio-demographic factors [age, gender (man/woman) and enrolment status (domestic vs. international student)].

Intention to use e-cigarettes in the future and susceptibility to use were dichotomised (yes/no) and a logistic regression performed to identify independent predictors of intention and susceptibility to e-cigarette use among those who had never used e-cigarettes. Covariates in the logistic regression model included variables from the univariate analysis comparing current and never use with a *p*-value less than 0.05 or with specific theoretical relevance to the analysis. As items assessing attitude to e-cigarette were correlated only one item was included “e-cigarettes are a gateway to smoking.”

For all tests a two-sided *p* < 0.05 was considered statistically significant.

## Results

A total of *n* = 1,094 responses were available for analysis. Eighteen respondents indicated their gender as gender diverse/non-binary (*n* = 18, 2.1%), the majority of respondents identified as woman (*n* = 536, 62.5%) and a small number preferred not to say (*n* = 13, 1.5%). Demographic characteristics and e-cigarette use among participants are presented in Table 1. International students (25% of the sample) were observed to be older than domestic students.

**Table 1:** Prevalence of e-cigarette use by demographic factors, serious psychological distress, alcohol use frequency, and weighted average mark.

		E-cigarette use			
	*N*	Daily	Current#	Past user	Never used
All participants	1,094	*N* = 80 (7.6%)	*N* = 138 (13.1%)	*N* = 145 (13.7%)	*n* = 772 (73.2%)
Age					
Mean (SD)	20.4 (1.9)	20.8 (1.9)^*^	20.8 (2.0)^**^	20.7 (2.0)^*^	20.3 (1.8)
Gender^					
Woman	536	*N* = 26 (4.9%)	*N* = 49 (9.1%)	*N* = 75 (14.0%)	*N* = 412 (76.9%)
Man	290	*N* = 33 (11.4%)^**^	*N* = 53 (18.3%)^**^	*N* = 34 (11.7%)	*N* = 203 (70.0%)
International student					
No	563	*N* = 35 (6.2%)	*N* = 71 (12.6%)	*N* = 77 (13.7%)	*N* = 415 (73.7%)
Yes	192	*N* = 15 (7.8%)	*N* = 22 (11.5%)	*N* = 23 (12.0%)	*N* = 147 (76.6%)
Serious psych distress					
No	829	*N* = 52 (6.3%)	*N* = 92 (11.1%)	*N* = 102 (12.3%)	*N* = 635 (76.6%)
Yes	117	*N* = 15 (12.9%)	*N* = 24 (20.7%)^**^	*N* = 18 (15.5%)	*N* = 74 (63.8%)
Alcohol use					
<Weekly	835	*N* = 47 (5.6%)	*N* = 77 (9.2%)	*N* = 88 (10.6%)	669 (80.2%)
>Weekly	191	*N* = 28 (14.7%)^***^	*N* = 52 (27.2%)^***^	*N* = 48 (25.1%)	91 (47.6%)
WAM^##^					
High (70+)	627	*N* = 35 (5.6%)	*N* = 66 (10.5%)	*N* = 26 (13.3%)	*N* = 473 (75.4%)
Low (<69)	195	*N* = 24 (12.3%)^**^	*N* = 39 (20.0%)^**^	*N* = 88 (14.0%)	*N* = 130 (66.7%)

^#^Daily, at least once a week, less than weekly. ^##^Self-reported Weighted Average Mark. ^Analyzed as a binary due to small *n* of genders other than woman/man. ^*^p < 0.05, ^**^p < 0.01, ^***^p < 0.001.

E-cigarette use was more prevalent than cigarette use. The proportion of people who use e-cigarettes daily (*n* = 80, 7.6%) did not differ between domestic and international students; however, men were more likely than women to report use of e-cigarettes (Table 1). Approximately 1 in 7 students (13.6%) reported current use (either daily, weekly or monthly use) of e-cigarettes and just over one quarter reported “ever use” of an e-cigarette (26.8%). E-cigarette use was greater among men, those reporting serious psychological distress, who used alcohol more frequently, and reported lower academic performance (Table 1).

Fruity flavored vapes were most commonly used (*n* = 104, 77.0%) followed by menthol/mint (*n* = 18, 13.0%). Nearly all people who reported current use, used e-liquids they believed contained nicotine (*n* = 110, 79.7%). Two thirds of students who used e-cigarettes daily reported using an e-cigarette on waking (*n* = 53, 66.3%).

Among all people who reported current use of e-cigarettes, the most common situations to use e-cigarettes were when hanging out with friends (81.2%), when drinking alcohol (60.9%) or when feeling stressed or anxious (56.5%) (Table 2). There were no differences between men and women respondents for situations where they would use e-cigarettes (data not shown); domestic students were more likely than international students to report using e-cigarettes at a party or club (Table 2).

**Table 2:** Situations where students are likely to use e-cigarettes.

	Current e-cigarette use *N* = 138	Domestic student *N* = 71^^^	International student *N* = 22^^^	*p*-value
	*n* (%)	*n* (%)	*n* (%)	*n* (%)
When hanging out with friends	112 (81.2%)	64 (90.1%)	16 (72.7%)	*p* = 0.040
When drinking alcohol	84 (60.9%)	51 (71.8%)	12 (54.5%)	*p* = 0.130
When I feel stressed or anxious	78 (56.5%)	46 (64.8%)	14 (63.6%)	*p* = 0.921
When I am bored or out of habit	68 (49.3%)	37 (52.1%)	10 (45.5%)	*p* = 0.585
In the morning when I first wake up	59 (42.8%)	29 (40.8%)	9 (40.9%)	*p* = 0.996
Other times	21 (15.2%)	10 (14.1%)	6 (27.3%)	*p* = 0.152
Preferred flavors				
Fruity	104 (77.0%)			
Menthol/mint	18 (13.0%)			
Tobacco	4 (2.9%)			
Coffee	1 (0.7%)			
Dessert/Creams	3 (2.2%)			
Other	5 (3.6%)			

^^^Numbers do not total 100% due to missing data.

Daily use of cigarettes, pipes or other tobacco products was uncommon (*n* = 34, 3.3%) although just more than 1 in 10 indicated they currently used any cigarettes (*n* = 120, 11.6%) with most use being among those who smoked less than weekly (*n* = 72, 7.0%). Dual use was common among people who used tobacco products daily (27/34, 79.4%) but less common among people who used e-cigarettes daily (27/76, 35.5%).

### Knowledge, attitudes and beliefs, social norms and self-efficacy

Mean e-cigarette knowledge was modest [3.02/5 (St Dev 1.10)] and there was no difference in knowledge between man and woman respondents (*p* = 0.426). Domestic students and people who used e-cigarettes currently tended to have higher scores for knowledge about e-cigarettes but these differences were not statistically significant.

More than half of respondents (51.2%) felt e-cigarette use was common within their peer groups however attitudes toward e-cigarettes and their impacts on health were predominantly negative (Table 3). Differences for seven out of nine statements about attitudes and social norms were found between people who reported current use compared to those who reported never use of e-cigarettes (Table 3).

**Table 3:** Perceived social norms, attitudes and beliefs toward e-cigarettes of participants.

	All participants	Current e-cigarette user	Never e-cigarette user	Test statistic	95%CI
	*N* = 1,094 (%)	*N* (%)	*N* (%)	(Exp(B))	
Vaping is common among my peer group					
Strongly disagree/disagree	368 (38.1%)	16 (13.2%)	352 (41.7%)	**REF**	
Neither/nor	103 (10.7%)	12 (9.9%)	91 (10.8%)	**3.027**	**1.231–7.441**
Agree/strongly agree	495 (51.2%)	93 (76.8%)	399 (47.5%)	**4.547**	**2.389–8.655**
E-cigarettes lower the risk of tobacco-related diseases					
Strongly disagree*/disagree*	503 (55.2)	51 (44.7%)	452 (56.6%)	REF	
*Neither/nor*	200 (21.9)	25 (21.9%)	175 (21.9%)	1.202	0.644–2.242
*Agree*/strongly agree	209 (22.9)	38 (33.3%)	171 (21.4%)	**2.445**	**1.455–4.109**
E-cigarettes are safer than regular cigarettes					
Strongly disagree*/disagree*	461 (50.9)	48 (42.5%)	413 (52.1%)	REF	
*Neither/nor*	196 (21.7)	27 (23.9%)	169 (21.3%)	1.442	0.794–2.619
*Agree*/strongly agree	248 (27.4)	38 (33.6%)	210 (26.5%)	**1.736**	**1.022–2.950**
E-cigarettes are less harmful to health than regular cigarettes					
Strongly disagree*/disagree*	484 (53.4)	48 (42.5%)	436 (55.0%)	REF	
*Neither/nor*	186 (20.5)	25 (22.1%)	161 (20.3%)	1.401	0.751–2.613
*Agree*/strongly agree	236 (26.0)	40 (35.4%)	196 (24.7%)	**2.247**	**1.334–3.784**
E-cigarettes are less harmful to the environment than regular cigarettes					
Strongly disagree*/disagree*	456 (50.2)	64 (56.6%)	392 (49.3%)	REF	
*Neither/nor*	241 (26.5)	26 (23.0%)	215 (27.0%)	0.671	0.380–1.185
*Agree*/strongly agree	211 (23.2)	23 (20.4%)	188 (23.6%)	0.751	0.424–1.330
E-cigarette aerosol is harmful for people in the vicinity of the user					
Strongly disagree*/disagree*	157 (17.3)	33 (29.2%)	124 (15.6%)	REF	
*Neither/nor*	210 (23.1)	31 (27.4%)	179 (22.5%)	0.610	0.322–1.156
*Agree*/strongly agree	541 (59.6)	49 (43.4%)	492 (61.9%)	**0.366**	**0.205–0.656**
E-cigarettes are a gateway to smoking					
Strongly disagree*/disagree*	184 (20.2)	37 (32.5%)	147 (18.4%)	REF	
*Neither/nor*	197 (21.6)	23 (20.2%)	174 (21.8%)	**0.510**	**0.263–0.989**
*Agree*/strongly agree	532 (78.4)	54 (47.4%)	478 (59.8%)	**0.401**	**0.233–0.690**
E-cigarettes are an effective way for smokers to decrease the number of cigarettes smoked (but not quit)					
Strongly disagree*/disagree*	227 (24.9)	21 (18.4%)	206 (25.9%)	REF	
*Neither/nor*	198 (21.8)	26 (22.8%)	172 (21.6%)	1.604	0.760–3.385
*Agree*/strongly agree	485 (44.3)	67 (58.8%)	418 (52.5%)	1.664	0.889–3.116
E-cigarettes are an effective way for people who smoke cigarettes to quit smoking					
Strongly disagree*/disagree*	398 (43.7)	34 (30.1%)	364 (45.7%)	REF	
*Neither/nor*	223 (24.5)	28 (24.8%)	195 (24.5%)	1.781	0.949–3.344
*Agree*/strongly agree	289 (31.8)	51 (45.1%)	238 (29.9%)	**2.372**	**1.371–4.104**

Model adjusted for gender (man/woman), age, international student status. Bolded figures indicate statistically significant differences between groups.

### Intention to use e-cigarettes

Among people who reported never using e-cigarettes, just under 1 in 4 respondents (*n* = 164, 22.8%) said they would use an e-cigarette if offered by a friend; 1 in 3 were curious about using e-cigarettes (*n* = 227, 31.5%) and *N* = 78 (10.8%) said they think they will try an e-cigarette soon. Nearly two in five [*n* = 279 (38.9%)] were considered susceptible to future use.

Results of logistic regression to determine independent predictors of intention to use e-cigarettes and susceptibility to use in the future is summarized in Table 4. Weekly or greater alcohol use (OR 4.805, 2.411–9.576) and low self-efficacy (OR 2.531, 1.061–6.037) were the strongest predictors of intention to use e-cigarettes among never users (Table 4). Women, participants reporting greater psychological distress, worse academic performance, or those with more positive attitudes toward e-cigarettes, and perception that vaping is common in their peer group were significant predictors of intention to use e-cigarettes in the future (Table 4).

**Table 4:** Logistic regression model of predictors of intention to use e-cigarettes and susceptibility to use of e-cigarettes in the future (never used).

	Intention to use			Susceptibility to use		
	Intention to use	Exp(B)	95% CI for Exp(B)	Susceptibility to use	Exp(B)	95% CI for Exp(B)
	Yes (*N* = 78)			Yes (*N* = 279)		
Alcohol use						
<Weekly	*N* = 55 (8.7%)	Ref		*N* = 232 (36.8%)	Ref	
Weekly or more	*N* = 23 (26.1%)	4.805	2.411–9.576	*N* = 47 (53.4%)	1.724	1.016–2.926
Self-efficacy						
High	*N* = 11 (4.8%)	Ref		*N* = 61 (26.8%)	Ref	
Low	*N* = 66 (13.6%)	2.531	1.061–6.037	*N* = 214 (44.3%)	1.509	0.999–2.281
Gender^^^						
Man	*N* = 17 (8.4%)	Ref		*N* = 73 (36.1%)	Ref	
Woman	*N* = 51 (12.4%)	2.329	1.154–4.700	*N* = 172 (41.8)	1.353	0.922–1.986
Attitude—e-cigarettes are a gateway to smoking						
Strongly agree/agree	*N* = 36 (8.8%)	Ref		*N* = 150 (36.8%)	Ref	
Neutral	*N* = 22 (14.5%)	2.288	1.77–4.864	*N* = 67 (44.4%)	1.512	0.922–2.480
Strongly disagree/disagree	*N* = 16 (13.7%)	2.057	1.040–4.067	*N* = 48 (41.0%)	1.364	0.879–2.117
WAM						
High	*N* = 44 (9.3%)	Ref		*N* = 184 (39.1%)	Ref	
Low	*N* = 22 (16.9%)	2.031	1.084–3.804	*N* = 59 (45.4%)	1.218	0.794–1.868
Vaping is common in my peer group						
[Less common (1)—more common (5)]	Mean = 3.40			Mean = 3.11		
	St Dev = 1.33	1.318	1.061–1.636	St Dev = 1.41	1.188	1.051–1.343
Kessler 6 total score						
	Mean = 14.07			Mean = 12.93		
	St Dev = 4.73	1.127	1.062–1.197	St Dev = 5.16	1.086	1.047–1.127

%, percentage within row. ^^^Analyzed as a binary due to small *n* of genders other than man/woman.

### E-cigarette health information sources

Students predominantly reported they sought health information about vaping from reputable, non-government health websites (77.3%), government reports/websites (72.9%), general practitioners (GPs) (67.9%), university health services (61.3%), or pharmacists (53.4%). Less reputable sources such as social media (30.7%), e-cigarette retailers (14.3%) and manufacturers (13.2%) were rarely nominated, however, those who did nominate them were significantly more likely to be an international student.

Most students indicated they would recommend friends or family members concerned about e-cigarette use to access reputable online resources such as Quit Victoria or Cancer Council Australia (*n* = 250, 27.7%) or their GP (*n* = 204, 22.6%). International students were least confident where to direct a family member or friend (15.9% selected “could not offer advice”) but the university health service was the most common reported service among international students (18.0%).

## Discussion

The prevalence of e-cigarette use in this cohort was much higher than previous studies of Australian university cohorts, but in line with increased community prevalence of e-cigarette use among young adults in Australia observed in community samples in the past 5 years. Current e-cigarette use was highest among those experiencing serious psychological distress, using alcohol more frequently, and with lower self-reported academic performance – all attributes that are likely to bring students into contact with university health services. Prevalence did not differ between domestic and international student groups which contrasts with a previous survey of Australian university students, and emphasizes the need to consider the needs of international students in health promotion or health service provision on campuses. More than one in three people who reported they had “never used” e-cigarettes were considered susceptible to future use and 1 in 10 intended to try an e-cigarette in the future. Levels of knowledge about e-cigarettes were modest. Mostly, students sourced information about health impacts of e-cigarettes from reputable online resources, or their GP, however, international students tended to rely more frequently on less reputable information sources including e-cigarette retailers and manufacturers and lacked confidence to direct friends or family who were concerned about e-cigarette use to appropriate supports.

Australia has seen a rapid increase in the use of e-cigarettes among adolescents in the past 5 years (1). This increase in community prevalence is reflected in the greater proportion of university students using e-cigarettes we identified compared with an earlier study of an Australian university cohort (20). Changes to the accessibility of e-cigarettes in Australia could impact upon use among university cohorts and the wider young-adult population more generally (38). Just under one in 10 of our respondents used e-cigarettes daily and provided indicators of addiction such as using e-cigarettes on waking. Care must be taken by the government as they adjust regulatory settings, to ensure this group are supported to quit use of nicotine, and not merely substitute nicotine from e-cigarettes to nicotine from other forms of tobacco products (39).

E-cigarette use in our sample was associated with a range of psychosocial and academic risks that may bring them into contact with health services consistent with previous reports (7, 40–42). In particular, psychological distress was more common among people who used e-cigarettes in our sample who also tended to use alcohol more frequently and reported worse academic performance which is consistent with findings from general population surveys of Australian adults (43). This highlights the importance of asking all students who present at health services about their smoking and vaping habits and discussing the associated harms. Many students may not voluntarily disclose their vaping use, despite using e-cigarettes as a coping strategy to manage stress (7). Students seeking support for stress, academic performance, or other general health counseling should be asked about e-cigarette use at every opportunity, and evidence-based treatments offered to these students together with behavioral support and referral where appropriate.

Addressing curiosity (44) and de-normalizing e-cigarette use, particularly in social activities, is crucial, and targeted public health campaigns that raise awareness of the potential harms of vaping could be effective in reducing intention to use e-cigarettes. The participants in this study overwhelmingly indicated they would source health information about e-cigarettes from reputable, online resources, however, international students, who make up approximately one third of the university student population, tended to rely on less reputable sources including social media, retailers, and manufacturers. Ensuring these students are aware of, and have access to, reputable sources of health information about e-cigarettes is important for this group and targeted strategies may be required for international students at Australian universities. Cultural variations in tobacco and nicotine consumption norms, as well as exposure to different nicotine control policies in their country of origin may impact upon their attitudes and beliefs to e-cigarette use (45, 46).

### Strengths and limitations

This study provides valuable insights into e-cigarette use among Australian university students and the health information sources young-adults use to inform their health decisions about vaping. International students comprised approximately one third of our sample and this is the first study to specifically consider their behaviors and health needs in relation to e-cigarettes. While our sample was over represented by women, the participation of international students was proportionally similar to, albeit it a little lower than, the general university student population.

Several limitations should be considered when interpreting the results of this study. The cross-sectional design means causal relationships between e-cigarette use and outcomes cannot be determined. We did not identify the degree/courses students were enrolled in so it is unclear if students with a greater health focus tended to participate, or whether the sample is broadly reflective of the range of course offerings available. Additionally, Monash University campuses are designated as smoking and vaping free, a policy that is known to be effective in reducing pro-tobacco beliefs, the acceptability of smoking, and decreases positive attitudes toward smoking (47). Among the respondents who used tobacco products we did not differentiate between those who smoked cigarettes, pipes or other tobacco products. Dual use as such, includes use of any of these types of tobacco products. Further, we did not ask about smokeless tobacco which can cause cancer, or nicotine pouches which are being increasingly promoted to young people in Australia by social media influencers.

About one third of the participants who started the survey did not complete it. We did not use imputation for missing data as there was no evidence of differences in prevalence of e-cigarette use between those who completed the survey and those who did not. Finally, *p*-values have not been adjusted for multiple comparisons, and so care should be taken in interpreting outcomes where there is risk of type 1 error.

## Conclusion

This study provides valuable and timely information about e-cigarette use and intentions to use e-cigarettes at a major Australian university. The high prevalence of e-cigarette use among both domestic and international students in our sample, and our finding that more than one in three respondents who had never used e-cigarettes were susceptible to future use, signals a need to address this issue with proactive preventive practices. Routine screening for e-cigarette use among young people who come in contact with university health services may be one appropriate example of this. Further research to understand how university students engage with health promotion messaging relating to e-cigarettes, the nature and forms of messaging most relevant to this group, including international student groups, is needed, to further inform future activities seeking to address e-cigarette use among university student cohorts.

## Data Availability

The raw data supporting the conclusions of this article will be made available by the authors, without undue reservation.
