# Supplementary material for: E-cigarette use and health information needs among a university student population in Melbourne, Australia
Source: Front Public Health. 2025 Apr 9;13:1563117. doi: 10.3389/fpubh.2025.1563117 (PMC12014626; doi:10.3389/fpubh.2025.1563117)
Supplement: Supplementary file 1 [file Data_Sheet_1.PDF]

## Intro

# Health information needs of young people in respect to vaping and e-cigarettes

You are invited to take part in a research study that involves completing a short, online survey from researchers at Monash University. The survey will take about 3 minutes to complete.

The aim of the study is to determine the knowledge, attitudes and health information needs of young people about e-cigarettes (or 'vapes') and their role in smoking cessation.

The findings from this study will help health care providers, like the University Health service, provide support and advice to young people about vaping and identify future areas of clinical need.

Participants who complete the survey are able to enter a prize draw to win 1 of 10 $100 e-gift cards. For full details about the study please download and read the explanatory statement here which includes contact details of the investigators and information about ethics committee approval of the study.

All responses are completely anonymous and so you will not be able to withdraw data once the survey is completed. By clicking next, you are consenting to take part in this study.

What is your age (years)?

- ○ 18
- ○ 19
- ○ 20
- ○ 21
- ○ 22
- ○ 23

- 24
- 25
- 26+

## Intro + vape use

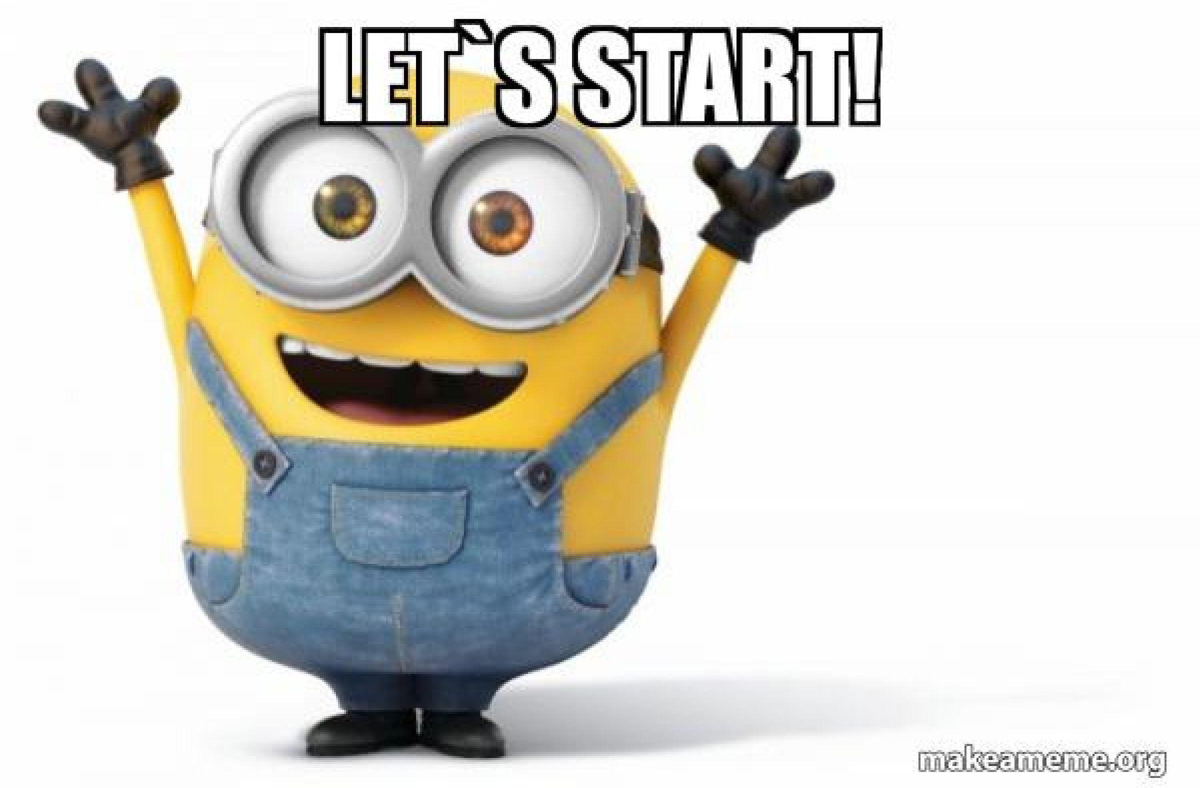


How often do you currently vape or use e-cigarettes?

- Daily
- At least once a week
- Less than weekly
- Not at all now, but I have been a regular e-cigarette user in the past
- Not at all now, but I have been an infrequent e-cigarette user in the past
- Not at all and I have never been a regular e-cigarette user

Do or did you own a vape?

- Yes

○ No

## Types of vapes

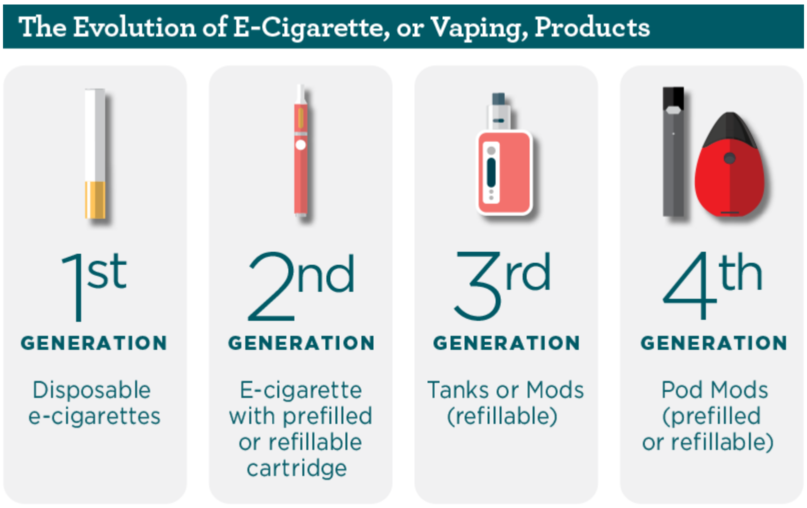


**https://www.zcisd.org/apps/pages/index.jsp?uREC_ID=364688&type=d&pREC_ID=2068845**

For example, disposable pod mods (4th generation) are currently the most popular type of vape.

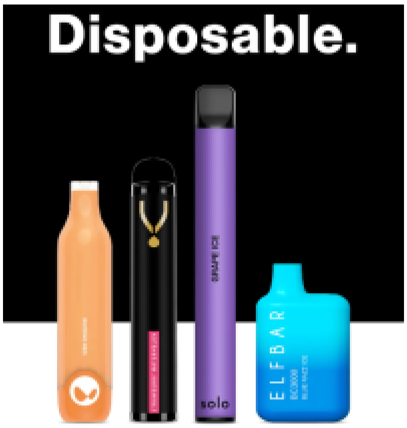


https://www.podlyfe.com.au/

## Which type of e-cigarette do/did you most commonly use?

○ 1st generation (e.g. cig-a-like e-cigarette)

○ 2nd generation (e.g. vape-pens, e-pens, e-cigars, e-hookahs)

○ 3rd generation (e.g. mod system, tank mods)

○ 4th generation (e.g. pod-based, JUUL)

○ I am not sure

What is/was your most preferred flavour?

- Fruity
- Menthol/Mint
- Tobacco
- Coffee
- Dessert/Creams
- Other

Do or did you use e-liquids (vape juice) containing nicotine?

- Yes
- No
- I am not sure

In which situations are/were you likely to vape / use e-cigarettes (select all that apply only)?

- In the morning, when I first wake up
- When hanging out with friends, e.g. at a party or club
- When drinking alcohol
- When I feel stressed or anxious
- When I'm bored or out of habit
- Other times

How often do you now smoke cigarettes, pipes or other tobacco products (does not include e-cigarettes or vapes)?

- Daily
- At least once a week
- Less than weekly
- Not at all now, but I have been a regular smoker in the past
- Not at all and I have never smoked

How often did you have a beverage containing alcohol in the past year?

| Never | Once a month or less | Once a fortnight | Once a week | 2-3 times a week | 4-6 times a week | Daily |
|---|---|---|---|---|---|---|
| ○ | ○ | ○ | ○ | ○ | ○ | ○ |

How many alcoholic beverages did you have on a typical day when you were drinking in the past year?

| None/I don't drink | 1 – 2 | 3 – 4 | 5 – 6 | 7 – 9 | 10+ |
|---|---|---|---|---|---|
| ○ | ○ | ○ | ○ | ○ | ○ |

How often did you have six or more alcoholic beverages on one occasion in the past year?

| Never | Less than monthly | Less than monthly | Weekly | Daily or almost daily |
|---|---|---|---|---|
| ○ | ○ | ○ | ○ | ○ |

Roughly during the past 30 days, about how often did you feel...

| | None of the time | A little of the time | Some of the time | Most of the time | All the time |
|---|---|---|---|---|---|
| Nervous? | ○ | ○ | ○ | ○ | ○ |
| Hopeless? | ○ | ○ | ○ | ○ | ○ |
| Restless or fidgety? | ○ | ○ | ○ | ○ | ○ |
| So depressed that nothing could cheer you up? | ○ | ○ | ○ | ○ | ○ |
| That everything was an effort? | ○ | ○ | ○ | ○ | ○ |
| Worthless? | ○ | ○ | ○ | ○ | ○ |

Your wellbeing is important to us. If you feel like you need to speak to a mental health professional, please contact a Monash Counsellor for free on **1300 788 336**. Support can be offered over the phone and in person and kept confidential. Alternatively, there are useful guidelines and resources about mental health and seeking help from BeyondBlue and the *Seeking Help* page.

## Attitude and beliefs

**Thinking about vaping in the community, your own use or the use of e-cigarettes by friends or family.**

Have you ever been curious about using e-cigarettes?

| Not at all curious | A little curious | Somewhat curious | Very curious |
|---|---|---|---|
| ○ | ○ | ○ | ○ |

If one of your best friends were to offer you an e-cigarette, would you use it?

| Definitely not | Possibly | Probably | Definitely yes |
|---|---|---|---|
| ○ | ○ | ○ | ○ |

Do you think you will try an e-cigarette soon?

| Definitely not | Possibly | Probably | Definitely yes |
|---|---|---|---|
| ○ | ○ | ○ | ○ |

Vaping is common amongst my peer group.

| Strongly disagree | Somewhat disagree | Neither agree nor disagree | Somewhat agree | Strongly agree |
|---|---|---|---|---|
| ○ | ○ | ○ | ○ | ○ |

I'm concerned about the use of e-cigarettes...

| | Not at all | A little | A lot |
|---|---|---|---|
| By others in the community. | ○ | ○ | ○ |
| By people I am close to. | ○ | ○ | ○ |
| My own use of e-cigarettes or vaping. | ○ | ○ | ○ |

**Self-efficacy is your belief in your own ability to control your behaviour and motivation in a range of different environments. This question assesses self-efficacy.**

I believe I can succeed at most of any endeavour to which I set my mind.

| Not true at all | Hardly true | Moderately true | Exactly true |
|---|---|---|---|
| ○ | ○ | ○ | ○ |

Which of the following statements about e-cigarettes in Australia do you believe is correct?

| | Yes | No | Unsure |
|---|---|---|---|
| Most contain nicotine. | ○ | ○ | ○ |
| Most contain tobacco. | ○ | ○ | ○ |
| They contain known carcinogens. | ○ | ○ | ○ |
| There is combustion (a burning process) | ○ | ○ | ○ |
| They are addictive | ○ | ○ | ○ |
| E-liquids (vape juice) containing nicotine can be purchased from pharmacies with a prescription from a doctor | ○ | ○ | ○ |
| E-liquids (vape juice) containing nicotine can be imported from overseas without a prescription | ○ | ○ | ○ |

**The following questions ask about your attitudes and beliefs about e-cigarettes and vaping. Please indicate your response for each question.**

| | Strongly disagree | Somewhat disagree | Neither agree nor disagree | Somewhat agree | Strongly agree |
|---|---|---|---|---|---|
| E-cigarettes lower the risk of tobacco-related diseases. | ○ | ○ | ○ | ○ | ○ |
| E-cigarettes are safer than regular cigarettes. | ○ | ○ | ○ | ○ | ○ |
| E-cigarettes are less harmful to health than regular cigarettes. | ○ | ○ | ○ | ○ | ○ |
| E-cigarettes are less harmful to the | ○ | ○ | ○ | ○ | ○ |

| | Strongly disagree | Somewhat disagree | Neither agree nor disagree | Somewhat agree | Strongly agree |
|---|---|---|---|---|---|
| environment than regular cigarettes. | | | | | |
| E-cigarette aerosol is harmful for people in the vicinity of the user. | ○ | ○ | ○ | ○ | ○ |

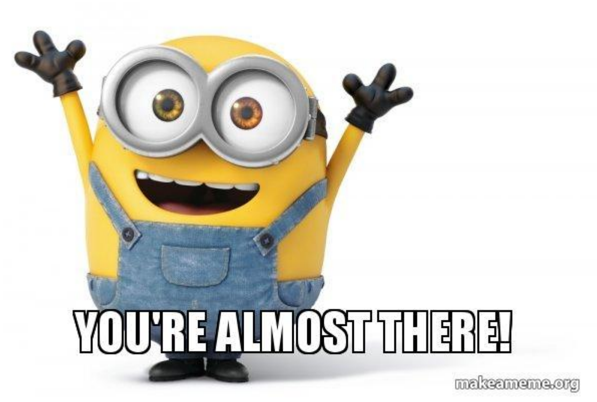


**NOT LONG TO GO NOW, JUST A FEW MORE QUESTIONS!**

**The following questions ask about your attitudes and beliefs about e-cigarettes and vaping. Please indicate your response for each question.**

| | Strongly disagree | Somewhat disagree | Neither agree nor disagree | Somewhat agree | Strongly agree |
|---|---|---|---|---|---|
| E-cigarettes are a gateway to smoking. | ○ | ○ | ○ | ○ | ○ |
| E-cigarettes are an effective way for people who smoke to decrease the number of cigarettes smoked (but not quit). | ○ | ○ | ○ | ○ | ○ |
| E-cigarettes are an effective way for people who smoke cigarettes to quit smoking. | ○ | ○ | ○ | ○ | ○ |

| | Strongly disagree | Somewhat disagree | Neither agree nor disagree | Somewhat agree | Strongly agree |
|---|---|---|---|---|---|
| I agree with the Australian Government's approach to e-cigarette policies and regulations. | ○ | ○ | ○ | ○ | ○ |

If a friend or family member asked you for help to quit vaping, where would you suggest they seek help ?

- ○ I could not offer any advice about using e-cigarettes, or vaping as I do not know enough about it.
- ○ I would suggest my friend not worry about it.
- ○ I would suggest the University Health Service could support them.
- ○ I would suggest their GP could support them.
- ○ I would suggest organizations like Quit Victoria or Cancer Council could support them.
- ○ I would suggest using a quit smoking app on a smart phone or device.
- ○ I would suggest they watch a YouTube video on vaping.
- ○ I would suggest they contact/find information from another person I know who has quit vaping.
- ○ I would not suggest any of these.

## Health information enquiries

Where would you seek health information about vaping or e-cigarettes?

| | I would access information about health effects of e-cigarettes from this source. | | |
|---|---|---|---|
| | Yes | No | Maybe |
| A GP | ○ | ○ | ○ |
| A pharmacist | ○ | ○ | ○ |
| The University Health Service | ○ | ○ | ○ |
| Government reports/websites | ○ | ○ | ○ |
| Websites from non-government health organisations (e.g. Quit Victoria, Cancer Australia, the Australian Lung Foundation) | ○ | ○ | ○ |
| Social media (e.g. TikTok, Instagram, Facebook) | ○ | ○ | ○ |

| | I would access information about health effects of e-cigarettes from this source. | | |
|---|---|---|---|
| | Yes | No | Maybe |
| Friends or family | ○ | ○ | ○ |
| E-cigarette retailers (Online retailers, stores/vape shops) | ○ | ○ | ○ |
| E-cigarette manufacturers | ○ | ○ | ○ |
| Other (please specify) ______ | ○ | ○ | ○ |

## Demographic questions

**To better understand the population we are surveying, please tell us a bit about yourself by answering the following questions.**

If you do not feel comfortable answering the questions below, you can choose to skip
.

What religious group do you belong to or identify yourself most close to?

- ○ Hindu
- ○ Jewish
- ○ Muslim
- ○ Christianity (Catholic protestant or any other Christian denominations)
- ○ I am not religious
- ○ Other (please specify) ______

**Ethnicity is a group you share your culture, language and traditions with.**

What ethnic group do you identify with most?

[dropdown]

What is your gender?

- ☐ Woman

- [ ] Man
- [ ] Non-binary / gender diverse
- [ ] My gender identity is not listed. I identify as: ____
- [ ] I prefer not to say

What is your Weighted Average Mark (WAM) approximately?

- 80-100 (High Distinction)
- 70-79 (Distinction)
- 60-69 (Credit)
- 50-59 (Pass)
- 0-49 (Fail)

I am enrolled at Monash University as a...

| Domestic student | International student |
| --- | --- |
| ○ | ○ |

Powered by Qualtrics
